# DNA barcoding identifies a cosmopolitan diet in the ocean sunfish

**DOI:** 10.1038/srep28762

**Published:** 2016-07-04

**Authors:** Lara L. Sousa, Raquel Xavier, Vânia Costa, Nicolas E. Humphries, Clive Trueman, Rui Rosa, David W. Sims, Nuno Queiroz

**Affiliations:** 1 CIBIO – Universidade do Porto, Centro de Investigação em Biodiversidade e Recursos Genéticos, Campus Agrário de Vairão, Rua Padre Armando Quintas, 4485-668 Vairão, Portugal; 2 Marine Biological Association of the United Kingdom, The Laboratory, Citadel Hill, Plymouth PL1 2PB, UK; 3 Ocean and Earth Science, University of Southampton, Waterfront Campus, European Way, Southampton SO14 3ZH, UK; 4 School of Biological Sciences, Cardiff University, Cardiff, CF10 3AX, UK; 5 Faculty of Sciences, University of Porto, Rua do Campo Alegre s/n, 4169-007 Porto, Portugal; 6 MARE – Marine and Environmental Sciences Centre, Laboratório Marítimo da Guia, Faculdade de Ciências da Universidade de Lisboa, Av. Nossa Senhora do Cabo, 939, 2750-374 Cascais, Portugal; 7 Centre for Biological Sciences, Building 85, University of Southampton, Highfield Campus, Southampton SO17 1BJ, UK

## Abstract

The ocean sunfish (*Mola mola*) is the world’s heaviest bony fish reaching a body mass of up to 2.3 tonnes. However, the prey *M. mola* consumes to fuel this prodigious growth remains poorly known. Sunfish were thought to be obligate gelatinous plankton feeders, but recent studies suggest a more generalist diet. In this study, through molecular barcoding and for the first time, the diet of sunfish in the north-east Atlantic Ocean was characterised. Overall, DNA from the diet content of 57 individuals was successfully amplified, identifying 41 different prey items. Sunfish fed mainly on crustaceans and teleosts, with cnidarians comprising only 16% of the consumed prey. Although no adult fishes were sampled, we found evidence for an ontogenetic shift in the diet, with smaller individuals feeding mainly on small crustaceans and teleost fish, whereas the diet of larger fish included more cnidarian species. Our results confirm that smaller sunfish feed predominantly on benthic and on coastal pelagic species, whereas larger fish depend on pelagic prey. Therefore, sunfish is a generalist predator with a greater diversity of links in coastal food webs than previously realised. Its removal as fisheries’ bycatch may have wider reaching ecological consequences, potentially disrupting coastal trophic interactions.

An important constraint in ecological studies, especially in the marine environment, is the lack of adequate knowledge on trophic interactions^1^. While direct observations of feeding events and examination of either gut or faecal contents have provided important information about marine food webs, these data have distinct shortcomings; for example they can significantly underestimate certain types of prey^2^. Analyses of stomach contents imply either the use of invasive stressful sampling procedures such as stomach flushing, or require examination of dead animals^3^,^4^. Moreover, such analyses rely on the correct identification of individual prey items which can be difficult after several hours of digestion^5^. While undigested individual prey items may be easily identified through morphology, consumed items very often consist of a pool of indistinguishable prey remains^6^. Stable isotope and fatty acid analyses provide an alternative view of animal diets e.g.^7^,^8^, but such biochemical approaches strictly trace the movement of structural nutrients between isotopically distinct trophic groups, and can only identify specific predator-prey interactions in very simple ecosystems. Recent advantages in genetic technology and global genetic databases allow species-level presence/absence information to be obtained from partially digested prey items recovered from animal stomachs and faecal samples^1^,^9^. The application of such DNA barcoding approaches in dietary studies of large predatory fish will likely reveal new and unexpected prey interactions and how these may change during ontogeny.

The ocean sunfish (*Mola mola* Linnaeus, 1758) is a widely distributed marine predatory fish but little is known about its ecology. As well as being the world’s heaviest bony fish, it is also the most fecund vertebrate, producing an estimated 300 million eggs^10^. The eggs are notably small (mean diameter 0.13 mm^11^) and so growth from a 0.25 cm larva to adult size is prodigious, requiring an increase in mass of 60 million times^11^. Nevertheless, the prey consumed by sunfish to provide energy for its impressive growth remains poorly understood. Until recently, *M. mola* was regarded as an obligate gelatinous zooplankton feeder^12^. This assumption, however, has only been supported by indirect evidence, such as fatty acid analysis of four individual sunfish caught off Nova Scotia^8^, and the co-occurrence of ocean sunfish and three jellyfish species (*Rhizostoma octopus*, *Chrysaora hysoscella* and *Cyanea capillata*) in the Irish and Celtic Seas^13^. Moreover, the small mouth gape of the sunfish compared for instance with that of the jellyfish-eating leatherback turtle, suggests a different type of prey other than large scyphozoan jellyfish^12^. Recent stable isotope analysis (SIA) of putative prey and muscle tissues from the dorsa of eight sunfish captured in the Mediterranean, challenged this view, revealing the possibility of different prey preferences and/or the occurrence of an ontogenetic shift in the diet^7^. The conclusions of this work raised some debate^14^,^15^ with the latter authors suggesting that the data was insufficient to disprove that sunfish are obligate consumers of gelatinous organisms^15^. Results from a recent work^16^, coupled with SIA and the visual identification of stomach contents from 17 sunfish captured off Japan, also suggest an ontogenetic shift in diet with age, with smaller sunfish feeding on benthic crustaceans, whereas larger individuals feed on gelatinous animals occurring in the water column. This preference in larger individuals for gelatinous prey was also confirmed by visual (animal-borne camera) recordings of feeding events on siphonophores, syphozoa and ctenophores^17^. Yet, to our knowledge, no studies have been undertaken to assess directly from stomach contents the apparently more generalist foraging strategy of this charismatic species using molecular tools.

In recent years, the emergence of next generation sequencing (NGS) has provided a powerful new approach to complex dietary studies^18^. This technology is responsible for an increasing number of studies using molecular barcodes to reveal diets of marine species such as fish, mammals and seabirds^3^,^19^,^20^. The implementation of barcoding approaches is, however far from straightforward^4^,^6^,^9^,^19^,^21^,^22^. While universal sets of primers are often used to maximize DNA detection from the widest possible range of prey species, they often enable the predator’s DNA to be co-amplified. As predator DNA is highly prevalent in gut contents it can be preferentially amplified over prey hampering barcoding efforts^19^. Thus, predator DNA removal techniques are highly recommended in diet studies using molecular barcodes^22^,^23^. A cost-effective technique is the use of predator specific non-extendable primers—the blocking primers—during the DNA amplification procedure. These specific primers are designed to overlap with the universal primers and to bind with the predator DNA, preventing its amplification^19^,^21^.

Within this context, the main objective of our study was to determine at the highest resolution possible (ideally at the species level), the dietary habits of the ocean sunfish in the north-east Atlantic Ocean, and thus determine its trophic position within food webs more accurately. For this we developed a strategy where universal PCR and predator-specific blocking primers were simultaneously used to successfully amplify barcode genes from 57 of the 100 digestive tract contents collected from sunfish.

## Results

**Figure 1:**
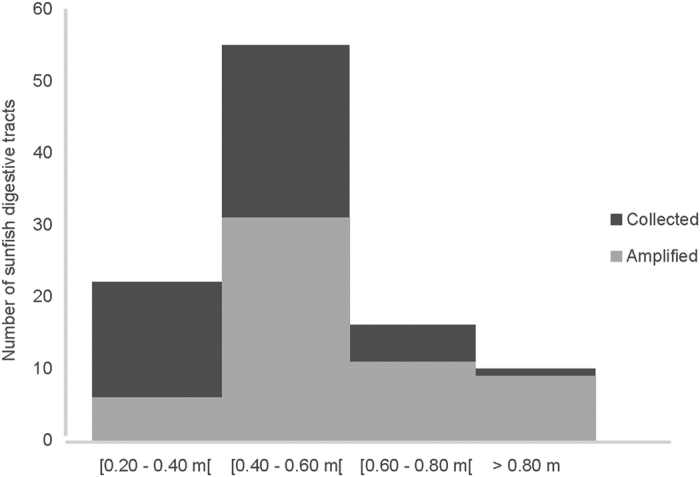
Individual sunfish sizes classes, from which digestive tracts were collected (black) and respectively amplified (grey).

Total length of the 100 sampled individuals ranged from 0.37 to 1.10 m, corresponding to juveniles and sub-adults^24^. The size distribution of captured fish is given in Fig. 1 and was representative of the length grouping of sunfish captured in the tuna set-net off southern Portugal: the majority of captured fish ranged from 0.40–0.60 m with larger fish being much less frequent. From the 100 sampled individuals, 57 were successfully amplified.

### Prey morphological identification

Besides the frequent visual identification of appendages of the swimming crab *Polybius henslowii*, further undigested prey items recovered comprised other crustaceans (e.g. GAMMARIDAE, COROPHIDAE and a megalopa larvae) and a *Conger* sp. larvae. In this study, undigested prey or body parts were found only in fish of sizes ≤0.60 m (size classes 1 and 2). In addition, cestode parasites were found in 63 intestines.

### Overview of sequencing data

We successfully amplified DNA from the digestive tracts of 57 sunfish (size class 1, *N* = 6; class 2, *N* = 31; class 3, *N* = 11; class 4, *N* = 9). All PCRs were sent for cloning and from these a total of 33 were successfully sequenced (totalling 119 clones). Despite this effort, only eight unique prey items were identified to species level, via cloning (Supplementary Table S1). A total of 5 clones were identified as sunfish, making up less than 5% of the total sequences. A second batch of PCRs were performed for NGS (approximately eight months later), and a total of 36 samples were successfully amplified. We presume that the decrease in amplification success was due to deterioration of prey DNA after consecutive freezing and defrosting cycles. A total of 75,091 DNA sequences were retrieved from the sequencing platform and the modal lengths for the amplicons received varied from 497 to 541 bp. Of these, 4152 (~5%) were identified in an initial blast as marine bacteria and were removed from the dataset. The final eukaryotic dataset comprised 70,939 sequences, of which sunfish made up a total of 3355 amplicons generated (less than 5%). A total of 39 unique prey were detected via NGS, of which six were also detected using cloning (Supplementary Table S1). Of these 39 prey items recovered via NGS, 14 were only identified after Statistical Assignment Package (SAP) processing.

### Sunfish prey items

**Figure 2:**
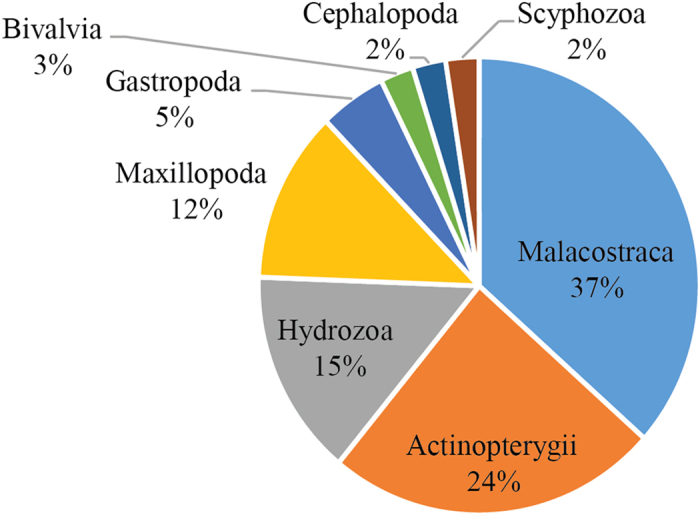
Summary of the prey taxa identified from sunfish consumed items.

A total of 41 unique prey items were identified genetically from the contents of sampled sunfish, via both cloning and NGS techniques, belonging to eight different taxonomic classes (Supplementary Table S1). Of these, 27 were found at the similarity level ≥98% using nucleotide sequence blast in GenBank and BOLD databases, and a further 14 prey items were subsequently found at 95% probability through Bayesian statistics in SAP. In total we found species from five Phyla and eight Classes. Overall, Malacostraca comprised ~37% of the sequences identified in the sunfish diet, Actinopterygii ~24%, followed by Hydrozoa at 15%. The remaining ~24% of the sequences included Maxillopoda, Bivalvia, Cephalopoda and Gastropoda (Fig. 2).

### Prey selection with respect to sunfish size class

**Figure 3:**
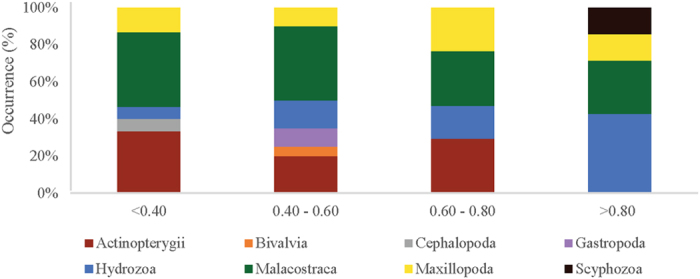
Barplot showing the occurrence of each taxonomic group consumed per sunfish size class (in metres).

The sequences identified from ocean sunfish gut contents sampled in this study were dominated by Malacostraca, Actinopterygii and hydrozoan prey. Interestingly, we found different dietary habits related to size (Fig. 3). Molecular data showed that for the sequences retrieved from sunfish larger than 0.80 m these are likely to have a more pelagic diet, comprising mainly gelatinous zooplankton species, a trend that was also evident from the digestive tract dissection as no hard parts (e.g. carapace, appendages) from other prey were present in larger fish. The number of sampled fish belonging to size class 4 (>0.80 m) was however smaller than for the remaining classes. The highest prey diversity was found in fish belonging to size class 2 (0.40–0.60 m), which was also the size class with more guts sampled (and was the predominant size class found within the tuna set-net). Specifically, small sunfish individuals (class 1 < 0.40 m) were found to consume prey belonging to five different taxonomic classes: Malacostraca (40%); Actinopterygii (33%); Maxillopoda (13%), Hydrozoa and Cephalopoda (7% each), whereas size class 2 individuals consumed prey from six different taxonomic classes: Malacostraca (40%), Actinopterygii (20%), Hydrozoa (15%), Gastropoda (10%), Maxillopoda (10%) and Bivalvia (6%). Sunfish of size class 3 (0.60–0.80 m) consumed four different taxonomic classes: Actinopterygii (29%), Malacostraca (29%); Maxillopoda (24%) and Hydrozoa (18%). In contrast, larger sunfishes (TL > 0.80 m) diet consisted of Hydrozoa (43%), Malacostraca (29%), Scyphozoa (14%) and Maxillopoda (14%). Percentages shown are relative to the total of prey items recovered for each size class.

The pelagic amphipod *Phrosina semilunata,* the siphonophore *Physophora hydrostatica*, the deep water fish *Hygophum benoiti* and the sea lice *Lepeophtheirus pollachiu* were found in fish from three out of the four size classes. Some other species, which were found to be common to at least two size classes were the crustaceans *Funchalia villosa*, *Pasiphaea sivado*, *Polybius henslowii*, *Liocarcinus holsatus*, the teleost *fish Conger conger*; and the hydrozoan *Rosacea cymbiformis*.

Finally, comparing diets across size classes using Bray–Curtis dissimilarity, ranging from 0 (no difference) to 1 (total difference), indicates that although the diet of smaller sized fish (below 0.80 m) was more diverse, dietary dissimilarities were lower (i.e., diets were more similar) between these size classes (for classes 1, 2 and 3: mean dissimilarity = 0.30) and higher between all three smaller size classes and the larger individuals (on average 0.54 dissimilar; Table 1).

## Discussion

One of the goals in studying the dietary habits of marine species, besides ascertaining its trophic position and predator-prey interactions, is to investigate habitat use and identify critical foraging habitats^25^. Here we showed that the diversity of prey taxa present in sunfish gut contents indicated that both benthic and pelagic food resources were taken by this species. DNA was successfully amplified from diet items of 57 out of 100 sunfish collected. We were able to assign barcoding sequences to a species or genus for 63% of the retrieved data. This high level of sequence recovery was similar to the levels of prey recovered using metabarcoding of the faeces of an omnivorous terrestrial mammal, the brown bear, using several group specific primer markers^20^. This suggests the methods we employed to investigate the diet of a large marine fish were at least as effective as those used more frequently to study terrestrial mammals.

In the current study a total of 41 different prey items were identified belonging to five different phyla, which confirms the recent proposals^13^,^16^ that, overall, the sunfish is a generalist predator. Moreover, the data also suggest an ontogenetic shift in the diet: while juveniles consumed teleost fish, Hydrozoa, Malacostraca, Maxillopoda, Bivalvia, Gastropoda and Cephalopoda prey, subadults seemed to be more selective, feeding mainly on Malacostraca, and hydrozoan and scyphozoan jellyfish. Altogether, our results contradict the classical view of sunfish being an obligate gelatinous zooplankton feeder, by supporting the findings of more recent studies^7^,^16^. Nonetheless, it is worth noting that the apparent lack of large-sized gelatinous zooplankton prey, the suggested preferred prey for sunfish^12^,^17^, may be due to the high digestibility of these soft-bodied animals and consequent rapid DNA degradation^26^. In fact, amplification of the siphonophore *Sulculeolaria quadrivalvis* occurred only at the first stage of this work, which suggests that DNA from these gelatinous prey was in fact degraded by the time the second stage was undertaken several months later. On the other hand, the fact that no adults >1.40 m were sampled could also help to explain these results, as predation on large syphozoa was only observed previously for larger individuals^17^. Smaller and coastal sunfish sampled in this study may not encounter large gelatinous zooplankton.

Ontogenetic shifts in the functional trophic group are ubiquitous among marine vertebrates^27^, including teleost species. Even though no adult fish were sampled in this study, we found evidence for an ontogenetic diet shift from juveniles to subadults, confirming the results found with SIA analysis performed elsewhere^7^,^16^. Interestingly, prey items belonging to the classes Malacostraca and Maxillopoda crustaceans were found in the diet of sunfish of all size classes, comprising 29–40% and 10–24% of the total sequences retrieved (sampled diet), respectively. While Actinopterygii were a key component of the diet of juveniles (representing between 20 and 30% of the total items found), they were absent from subadults (>0.80 m). Similarly, Cephalopoda, Bivalvia and Gastropoda were only consumed by juvenile sunfish of smaller sizes (classes 1 and 2: 0.40–0.60 m), while cnidarians including hydrozoans and scyphozoans were more prevalent in subadult diets (class 4: >0.80 m), totalling about 57% of retrieved sequences. Furthermore, the Bray–Curtis index revealed a greater similarity between the diet compositions of different size classes of juveniles when compared to subadults. Smaller individuals seem to have a broader trophic niche than larger fish, probably feeding more opportunistically on what is available in the shallow coastal, bentho-pelagic habitats they occupy.

The presence of coastal prey in smaller sunfish, such as the euphausiid *Thysanoessa gregariai*, the burrowing shrimp *Jaxea nocturna*, the northern krill *Meganyctiphanes norvegica* of shelf/slope waters, the benthic crab *Goneplax rhomboids*, and the mussel *Mytilus galloprovincialis* suggests a juvenile preference for coastal habitats^28^,^29^. In addition, all the teleost species found to be consumed by juveniles are known to spawn along the coasts of the Gulf of Cadiz and western Iberia in a time frame that is coincident with our sampling, or are known to remain in shallow coastal waters for long periods of time, e.g. the myctophid *Hygophum benoiti*^30^; blue jack mackerel *Trachurus picturatus*^31^; gilthead seabream *Sparus aurata*^32^ and the conger eel *Conger conger*^33^. Besides being able to feed on the benthos (see video recordings in^16^), our data also revealed the presence in sunfish diet of the mesopelagic pearlside *Maurolicus muelleri*, the decapod *Pasiphaea sivado*, and the swimming crab *P. henslowii*^34^,^35^, indicating that the small sunfish might also be actively feeding in the water column. In the case of large-sized sunfish (class 4), all identified prey species were pelagic: the decapod crab *P. henslowii*, the siphonophore *Physophora hydrostatica*, the likely indirect consumption of the amphipod *Phrosina semilunata* known to be associated to gelatinous zooplankton for transportation and protection within the pelagic waters^36^, the epiplanktonic nectophore *Rosacea cymbiformis*^37^ and the siphonophore *Sulculeolaria quadrivalvis*^38^. Moreover, the single scyphozoan taxa (also pelagic) detected was found in individuals larger than 0.80 m. Hence, the exclusive incidence of pelagic fauna seems to confirm the dependency of larger sunfish on the water column to feed^17^.

We acknowledge other possible contributors to sunfish gut content DNA could confound the results obtained here^23^, including the presence of ectoparasites (e.g. *Caligus*) and/or the secondarily ingested organisms. The broad occurrence of *Caligus* in all four classes suggest that these organisms could have been ingested by sunfish consuming prey with parasites that were common to all individual size classes, or, have opportunistically penetrated dead sunfish prior to sampling. Similarly, the well-established consumption of eggs and larvae of teleosts by gelatinous species^39^ could also confound the obtained results (secondary predation or hyperpredation). However, in this study the visual identification of an undigested *Conger spp* larvae, freshly consumed by the sunfish, confirms the predation on other teleost species. Hence, despite recognising the possibility for including secondary prey in sunfish digestive tract contents as primary prey items, there was no clear evidence to exclude any item found from having been directly consumed.

The prevalence of small-sized sunfish in the tuna set-net is consistent with the results from a recent satellite tracking study of this species in the north-east Atlantic, where a more coastal occurrence of smaller sized fish was detected when compared to larger individuals^40^. Moreover, the same study described the occurrence of both normal and reverse diel vertical migration (DVM) together with surface oriented and an irregular behavioural pattern in the depth occupancy of subadult sunfish individuals. These diverse vertical movement patterns seem to conform to the behaviour of opportunistic feeding on prey where they are most available. The study of Nakamura *et al.*^17^ focusing on larger sunfish (>1.40 m), showed that these individuals responded to patchily distributed prey in the water column, exhibiting the DVM behaviour found for other gelatinous predators e.g.^41^.Taken together with our dietary data, these vertical movement patterns likely reflect the foraging habits of a generalist predator.

As expected, our results support the superior performance of NGS compared with cloning techniques. Besides the technical difficulties inherent to cloning (e.g. transforming competent cells), accurate diet characterisation depends on the number of clones selected for sequencing. Nevertheless, some studies have been successful in assessing diet of marine organisms using cloning, for example in the case of the Steller sea lions (*Eumetopias jubatus*)^42^, where cloning was used to test the efficiency of DNA-based methods in diet reconstructions, and for the Australian sea lion, where 28 prey items were identified using cloning^25^. Furthermore, the use of cloning even provided enough resolution to detect ontogenetic shifts in diet in the case of the largemouth bass (*Micropterus salmoides*), from which 26 prey species were retrieved^1^. In the present study, cloning allowed the identification of two prey species, the fish *Scomber colias* and the hydrozoan *Sulculeolaria quadrivalvis*, which were not detected by NGS. However, this could be due to the degradation of DNA following the freezing and defrosting after the first PCR reaction (for cloning). Another explanation is that different PCR reactions amplified DNA from different prey species due to the known possible bias of primers at binding sites, which could make some prey species more prone to amplification than others e.g.^43^. In our study for the same sample, we included as many repetitions as possible of PCR products which were then purified and quantified before sent for NGS.

Typically, generalist predators play a key role within food webs and their presence is usually related to strong top-down forcing^44^. Yet, generalist predation is also known to regulate other trophic levels in ways not predicted by cascading trophic interactions^45^. Thus, in the light of the present results, the persistence of incidental captures of the cosmopolitan sunfish worldwide^46^,^47^,^48^,^49^ may have important and wider implications for marine ecosystems than previously thought. Although quantifying bycatch is difficult, some studies documented that sunfish totalled 70 to 93% of the fish caught in Spanish drift-gillnet fisheries within the Mediterranean between 1992–1994^47^, and that it represented 51% of all bycatch in the Cape horse mackerel midwater trawl fishery in South Africa^46^. In some regions of the world, this trend is changing suggesting that local population sizes might be decreasing^46^. Importantly, recent reassessment made by the International Union for the Conservation of Nature (IUCN) Red List Threatened Species considered sunfish to be facing a high risk of exploitation and the species’ conservation status was updated to Vulnerable^50^. This is especially concerning as genetic analyses suggest the existence of several isolated sunfish populations rather than a single global population^51^. Furthermore, restricted regional movements have been described for the species^49^,^52^ and hence this species may be more vulnerable to local depletion than previously thought. Importantly, our results help to clarify the trophic position of this charismatic species which may prove useful for informing data-driven assessments of its habitat preferences and, consequently, its potential vulnerability to fisheries exploitation.

## Methods

### Sunfish digestive tract collection and pre-processing

We collected gut contents of 100 individuals within a set-net targeting tuna, off Olhão, southern Portugal, where dozens of healthy sunfish are captured and released daily. No live vertebrates were involved in our experiments. Occasionally, due to either atmospheric and/or oceanographic conditions, daily fishing operations are disrupted which may result in the death of some trapped fish. Our sampling targeted such freshly dead fish and occurred during April–June (spring) months of 2013 and 2014, with 50 individuals being sampled each year. No evidence of differences in either individual sizes or visual identification of the consumed items were recorded between sampling years. Digestive tracts (stomach and intestines) from each individual were collected during fishing operations and maintained on ice while on-board (<1 hour). In the lab stomach and gut contents were immediately transferred to 200 mL universal containers and kept at −20 °C until further processing. Undigested remains from gut contents were stored in 96% ethanol and were morphologically identified and photographically recorded through a dissecting microscope. However, most of the gut contents consisted of completely digested remains, which were manually homogenised before a 2 mL subsample was taken for DNA extraction.

### DNA Extraction and amplification

Genomic DNA was extracted using the JetQuick DNA Kit (GENOMED), following the protocol for purification of total DNA from animal tissues, with some adaptations. The first change to the standard protocol was the volume of reagents used to digest the recovered content: a total of 4 ml of lysis buffer was added to 2 ml of gut content of each individual, along with 60 μl of proteinase K, in a 15 ml tube; the samples were incubated overnight at 56 °C. Due to the initial large volume, several repetitions of the following steps of the protocol had to be performed. In addition, DNA from sunfish muscle and that of the pelagic crab, *Polybius henslowii*, which was frequently observed in the gut contents, was also extracted, following the standard protocol, and were used as controls for the subsequent polymerase chain reactions (PCR). Extracted DNA was immediately frozen at −20 °C until PCR was carried out.

Despite being controversial, the universal COI (cytochrome oxidase subunit I) primers are still recommended as a metabarcoding marker, particularly when species-level identification is critical, as in our study^53^. Hence, we used degenerate versions of the COI primers LCO1490 and HCO2198^54^, the jgLCO1490 (5′ TITCIACIAAYCAYAARGAYATTGG 3′) and the jgHCO2198 (5′ TAIACYTCIGGRTGICCRAARAAYCA 3′)^55^. To quantify the amplification of sunfish DNA, PCR was performed for a random subset of 10 individuals and sequencing through cloning (10 clones per individual). Since over 60% of the sequences retrieved belonged to the sunfish, we opted to design a blocking primer 5′-CAAAGAATCAGAAGAGATGTTGA [SpcC3]-3′ based on the mitochondrial genome of sunfish available on GenBank (Accession number AP006238). This primer overlapped with the 3′ end of the reverse universal primer, but extended into the sunfish specific sequence, and was modified with a C spacer following 33, to prevent elongation without affecting the annealing properties. Additionally, we split the samples into four different fish size classes (<0.40 m; 0.40–0.60 m; 0.60–0.80 m and >0.80 m TL), to better characterise the sunfish diet with growth and to detect possible changes in the dietary habits. Thus, in PCRs to be sequenced through NGS we used four different multiplex identifiers from Roche (MIDs) attached to the original primer sequences (MID001: ACGAGTGCGT; MID002: ACGCTCGACA; MID003: AGACGCACTC; MID004: AGCACTGTAG).

For DNA amplification, 1 μl of the extracted DNA was added to a total of 19 μl of PCR reaction mix and proof reading Platinum Taq (Invitrogen) was employed in all PCR reactions (see Supplementary Table S2 for PCR mix descriptions). Firstly, we tested the efficiency of two different concentrations of blocking ^primer^ [10 and 20x] relative to the universal COI primers, to prevent sunfish DNA amplification during PCR. We found that a proportion of 20:1 of blocking primer was needed to ensure minimum sunfish DNA amplification.

A touchdown PCR amplification protocol was implemented due to the higher temperature demand of the blocking primer to bind to the sunfish DNA. Thermal cycling conditions were as follows: initial denaturation at 95 °C for 5 minutes followed by 62 cycles of: 95 °C for 30 seconds; annealing temperature step-downs every 4 cycles of 1 °C (from 55 °C to 47 °C) until the temperature reached 46°C which was maintained for 20 cycles; 72 °C for 30 seconds; and a final extension at 72 °C for 7 minutes. DNA amplification of the contents and *P. henslowii*, and absence of amplification for the sunfish DNA and PCR reagents negative control (blank), were verified in an agarose gel (e.g. Supplementary Fig. S1). PCR reactions used in NGS were repeated at least three times per sample, and were pooled as explained in the next section.

### Cloning and NGS

A total of 38 PCRs were sent to an external service for cloning (Centro de Testagem Molecular - CTM; CIBIO InBIO-UP) and on average 10 clones per sample were sequenced. For the 96 PCRs prepared for NGS, purification was accomplished using Agencourt AMPure XP following the manufacturers’ protocol. Final purified PCR product was eluted in 28 μl of water. DNA quantification was performed using Quant-iT PicoGreen dsDNA Assay Kit. Samples for which DNA concentration did not exceed the 10 ng/μl, the minimum requested for NGS proceeding, were concentrated using Speed Vac concentrator Eppendorf at room temperature, during cycles of 45 minutes, until a final volume of ca. 13 μl. Finally, PCR products were pooled and sent to Beckman Coulter Genomics to be sequenced by a Roche 454 GS FLX Sequencing Platform.

### Data treatment

#### Identification of sequences retrieved through cloning

We performed Basic Local Alignment Search Tool BLAST - searches against sequences present in the NCBI GenBank (http://www.ncbi.nlm.nih.gov/) and BOLD databases (http://www.boldsystems.org/index.php/). All possible genetic codes (Invertebrate; Vertebrate; Echinoderms; Ascidian) were investigated to ensure the best translation possible. Additionally, sequences were translated into protein and protein BLAST searches were performed to discard pseudo genes. Resultant identification at species level was only accepted if the sequence similarity with best match exceeded the 97%^56^.

#### Metabarcoding (NGS) sequences treatment: Filtering, alignment, clustering and taxonomic assignment

We obtained a 454 platform output “standard flowgram file” demultiplexed by MID (sunfish size class). Next, a Pyronoise procedure was implemented using the *mothur* software package version 1.35.1^57^ and following the standard operating procedure (SOP) to process sequences generated by 454 pyrosequencing (http://www.mothur.org/wiki/454_SOP). Briefly, only sequences longer than 300bp and unique haplotypes were retained for further analysis. Additionally, whenever more than 8 homopolymers (likely sequencing errors) were present in sequences these were discarded. Subsequently, following Ranwez *et al.*^58^, Macse v1.01b was then implemented to translate automatically nucleotide into amino acid sequences and perform BLAST searches within GenBank, using all possible genetic codes. A twofold downstream analysis was performed: (a) nucleotide sequences were blasted to firstly remove sequences with stop codons [bacteria or pseudo genes], chimeric sequences and frame shifts [presumably caused by errors during the 454 platform sequencing procedure^19^], together with sequences with similarity of 97% or lower^19^,^56^; (b) amino acid translated sequences were also taxonomically assigned to the lowest taxonomic group according to the homology attained per sequence. Further detailed description of the methodology employed can be found in the supplementary material. We found this approach useful to detect errors in the nucleotide Genbank database, namely bacterial sequences mislabelled as fish and other marine organisms^59^. Furthermore, we also used the BOLD database to compare our sequences and further confirm the results with GenBank.

Lastly, a Bayesian approach implemented in the Statistical Assignment Package (SAP)^60^ that uses the Genbank reference database, was employed to assign those sequences with lower similarity scores (<98%) to the highest taxonomic rank possible. Briefly, the posterior probability of a nucleotide sequence belonging to a specific taxonomic rank represented in Genbank database was calculated by building 10,000 phylogenetic trees. To do so, a total of 50 homologue sequences with similarity scores higher than 70%, were downloaded from Genbank and taxonomic assignments were only made at significance level equal or higher than 95%^60^. Further details may be found in the supplementary material.

#### Prey identification per sunfish size class: Quantifying diet composition and overlap

To investigate the differences in diet composition and overlap among the four different size classes we measured the prey variability among each class using the Bray–Curtis dissimilarity index implemented in the ‘vegan’ package in R software. This rank-order similarity index was then applied to prey taxa grouped by sunfish size class.

## Additional Information

**How to cite this article**: Sousa, L. L. *et al.* DNA barcoding identifies a cosmopolitan diet in the ocean sunfish. *Sci. Rep.* **6**, 28762; doi: 10.1038/srep28762 (2016).

## Supplementary Material

Supplementary Information

## Figures and Tables

**Table 1:** Bray-Curtis dissimilarity matrix (weighted means) for diet composition among different sunfish size classes (low values = high overlap).

	**<0.40 m**	**0.40–0.60 m**	**0.60–0.80 m**
0.40–0.60 m	0.31		
0.60–0.80 m	0.28	0.29	
>0.80 m	0.56	0.58	0.48
