# Supplementary material for: DNA barcoding identifies a cosmopolitan diet in the ocean sunfish
Source: Sci Rep. 2016 Jul 4;6:28762. doi: 10.1038/srep28762 (PMC4931451; doi:10.1038/srep28762)
Supplement: Supplementary Information [file srep28762-s1.doc]

**Supplementary Information for:**

**DNA barcoding identifies a cosmopolitan diet in the ocean sunfish**

Lara L. Sousa^1,2,3^, Raquel Xavier^1,4^, Vânia Costa^1,5^, Nicolas E. Humphries^2^,Clive Trueman^3^, Rui Rosa^6^, David W. Sims^2,3,7^, Nuno Queiroz^1*^

**Supplementary methods:**

***Treatment of metabarcoding sequences - Denoising of sequences***

A 454 platform standard flowgram output file per sunfish size class was obtained from Beckman Coulter Genomics and after separating bacterial nucleotide sequences, the remaining bulk was filtered as follows:

1. Pyronoise procedure implemented in Mothur: a) trim.seqs function was used to remove the known mid tags barcode and primer sequences, while also rejecting all sequences with length smaller than 300 bp long and with homopolymers longer than 8 bp; b) unique.seqs was further implemented to simplify the dataset, by removing duplicates, leaving only the unique sequences; and lastly c) chimera.uchime was applied to the unique sequences screening for sequences formed from two or more biological sequences joined together (chimeras – thought to arise from incomplete extension during PCR). Chimeric sequences were removed from the pool.

2. Filtering amino acid sequences in *Macse*: Nucleotid sequences retrieved from mothur procedure were then translated into protein sequences using Macse v1.01b [^58^](#_ENREF_58). Macse can detect interruptions in open reading frames, either caused by nucleotide substitutions that result in stop codons or the insertion or deletion/nucleotides that induce frameshifts[^21^](#_ENREF_21). Thus, an automatic translation was performed while rejecting sequences with stop codons likely to belong to bacteria or pseudo genes and with frameshifts which presumably result from errors during the 454 platform sequencing procedure.

*Taxonomic assignment of filtered sequences*

GenBank is a comprehensive public database of DNA sequences, supporting bibliographic and biological annotation, built and distributed by the National Centre for Biotechnology Information (NCBI), USA. Each GenBank record consists of both a sequence and its annotations and is assigned a stable unique identifier, the accession number. BLAST is a search engine for sequence-similarity against a sequence database, such as GenBank, which works by locating regions of similarity between the sequence of interest and the database records. BLAST searches were performed on NCBI’s web site (http://blast.ncbi.nlm.nih.gov/Blast.cgi) and received alignments had associated scores and measures of statistical significance. Here, we used the GenBank database BLAST service to classify our filtered (a) nucleotide sequences, accepting the assigned taxon to those sequences with similarity higher than 98%; (b) aminoacid translated sequences assigned to the lowest taxonomic group possible, given the homology attained per sequence.

For those sequences with BLAST similarity below the threshold of 98% we applied a Bayesian approach that allowed us to estimate the probability for the sample sequence to belong to a monophyletic group, identified with GenBank database sequences. Hence, we used the Statistical Assignment Package (SAP)[^60^](#_ENREF_60), a Bayesian classifier, to classify each sequence to GenBank reference database by building 10,000 phylogenetic trees and calculating the posterior probability, via Markov Chain Monte Carlo (MCMC), that the query species belong to a particular taxonomic group, for all levels of taxonomic annotation. This posterior probability enables the assignment of the sequence to a higher ranking taxon, when homology information is too ambiguous at the species level[^60^](#_ENREF_60). In summary, we permitted SAP to download 50 GenBank homologues at ≥ 70% sequence identity and accepted retrieved assignments only at the significance level of 95% (posterior probability) from the phylogenetic trees, at the respective lower taxon possible.

**Supplementary Table S 1** List of individual prey items identified through barcoding, with accession number from GenBank database, Taxonomic Class and sequencing method.

| **Size Class** | **Accession** | **Class** | **Lowest taxonomic level** | **Method** | **Similarity** | **Bayesian Probability** |
| --- | --- | --- | --- | --- | --- | --- |
| **1, 2 and 3** | HQ167651 | Actinopterygii | *Hygophum benoiti* | NGS | ≥98% | - |
| **1, 2 and 4** | EF989670 | Malacostraca | *Phrosina semilunata* | NGS/cloning | ≥98% | - |
| **1, 3 and 4** | GQ120035 | Hydrozoa | *Physophora hydrostatica* | NGS/cloning | ≥98% | - |
| **1 and 2** | JQ306133 | Malacostraca | *Funchalia villosa* | NGS | ≥98% | - |
| **1, 2 and 3** | AY861363 | Maxillopoda | *Lepeophtheirus pollachius* | NGS/cloning | ≥98% | - |
| **1 and 3** | JQ775007 | Actinopterygii | *Conger conger* | NGS/cloning | ≥98% | - |
| **1 and 3** | JQ306263 | Malacostraca | *Pasiphaea sivado* | NGS | ≥98% | - |
| **1 and 3** | * | Maxillopoda | *Caligus (*genus*)* | NGS | ≥70% | ≥95% |
| **1 and 3** | * | Actinopterygii | *Teleostei (*infraclass*)* | NGS | ≥70% | ≥95% |
| **2 and 3** | * | Hydrozoa | *-* | NGS | ≥70% | ≥95% |
| **2 and 3** | GQ268538 | Malacostraca | *Liocarcinus holsatus* | NGS | ≥98% | - |
| **2 and 4** | GQ120042 | Hydrozoa | *Rosacea cymbiformis* | NGS/cloning | ≥98% | - |
| **3 and 4** | JQ306039 | Malacostraca | *Polybius henslowii* | NGS/cloning | ≥98% | - |
| **3 and 4** | KF483708.1 | Maxillopoda | *Caligus sclerotinosus* | NGS | ≥70% | ≥95% |
| **1** | HM593055 | Actinopterygii | *Scomber japonicus* | NGS | ≥98% | - |
| **1** | KC015306 | Actinopterygii | *Cubiceps gracilis* | NGS | ≥98% | - |
| **1** | KC860970 | Cephalopoda | *Mastigoteuthis atlantica* | NGS | ≥98% | - |
| **1** | AY047604 | Malacostraca | *Thysanoessa gregaria* | NGS | ≥98% | - |
| **1** | FJ581756 | Malacostraca | *Meganyctiphanes norvegica* | NGS | ≥98% | - |
| **1** | EF989676.1 | Malacostraca | *Cystisoma pellucida* | NGS | ≥70% | ≥95% |
| **2** | KC409639 | Actinopterygii | *Sparus aurata* | NGS | ≥98% | - |
| **2** | KJ709658 | Actinopterygii | *Trachurus picturatus* | NGS | ≥98% | - |
| **2** | HM007765 | Actinopterygii | *Lophius (*genus*)* | NGS | ≥98% | - |
| **2** | DQ864410 | Bivalvia | *Mytilus galloprovincialis* | NGS | ≥98% | - |
| **2** | KC774030 | Gastropoda | *Cavolinia inflexa* | NGS | ≥98% | - |
| **2** | JQ306144 | Malacostraca | *Jaxea nocturna* | NGS | ≥98% | - |
| **2** | JQ305932 | Malacostraca | *Plesionika narval* | NGS | ≥98% | - |
| **2** | JN591694 | Malacostraca | *Goneplax rhomboides* | NGS | ≥98% | - |
| **2** | FJ876945.1 | Gastropoda | *Pneumoderma violaceum* | NGS | ≥70% | ≥95% |
| **2** | AY386273.1 | Maxillopoda | *Caligus elongatus* | NGS | ≥70% | ≥95% |
| **2** | GQ119963.1 | Hydrozoa | *Ceratocymba (*genus*)* | NGS | ≥70% | ≥95% |
| **2** | * | Malacostraca | *Euphausiidae (*family*)* | NGS | ≥70% | ≥95% |
| **2** | * | Malacostraca | *Polybiidae (*family*)* | NGS | ≥70% | ≥95% |
| **3** | EU148247 | Actinopterygii | *Maurolicus muelleri* | NGS | ≥98% | - |
| **3** | AB488406 | Actinopterygii | *Scomber colias* | cloning | ≥98% | - |
| **3** | KC287552 | Maxillopoda | *Clausocalanus furcatus* | NGS | ≥98% | - |
| **3** | * | Malacostraca | *Nyctiphanes (*genus*)* | NGS | ≥70% | ≥95% |
| **3** | AY937370 | Hydrozoa | *Forskalia tholoides* | NGS | ≥70% | ≥95% |
| **3** | KC545792 | Malacostraca | *Solenocera crassicornis* | NGS | ≥70% | ≥95% |
| **4** | * | Scyphozoa | *-* | NGS | ≥70% | ≥95% |
| **4** | GQ120050 | Hydrozoa | *Sulculeolaria quadrivalvis* | cloning | ≥98% | - |

*No accession number is given if probability of belonging to a certain species is lower than 95% threshold.

**Supplementary Table 2** PCR reaction mix per individual (20 µl).

|  | **Cloning** | **NGS** |
| --- | --- | --- |
| **H20** | 12.25 | 11.80 |
| **PCR Buffer** | 2.08 | 2.00 |
| **MgCl2 (50mM)** | 1.04 | 1.30 |
| **dNTP mix** | 0.41 | 0.40 |
| **Primer jgLCO1490** | 0.82 | 0.80 |
| **Primer jgHCO2198** | 0.82 | 0.80 |
| **Blocking primer** | 1.63 | 1.60 |
| **Taq Polymerase** | 0.16 | 0.20 |
| **Bovine Serum Albumin** | 0.10 | 0.10 |


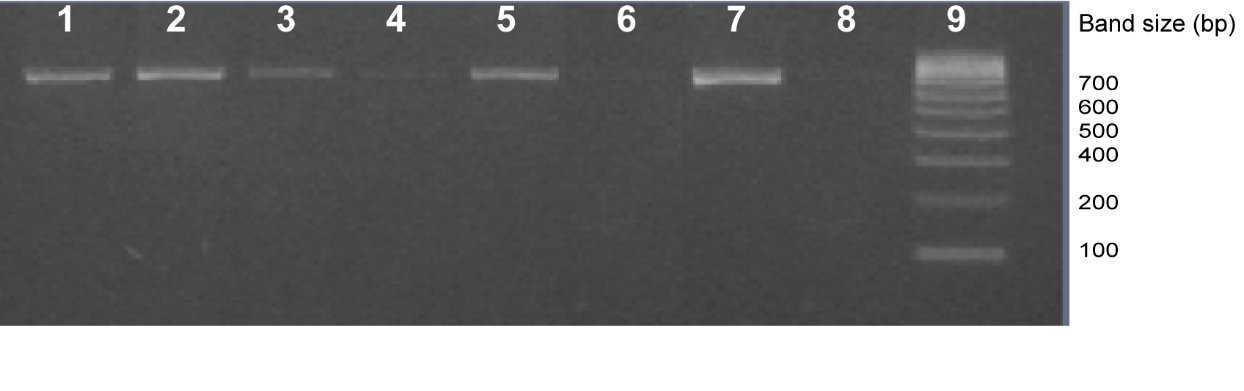


**Figure S 1** PCR confirmation of the efficiency of ocean sunfish specific blocking primer. Gel lanes correspond to: prey item amplified DNA (1-5); sunfish DNA (6); *P. henslowii* DNA – positive control (7); reaction negative control (blank) (8) and molecular ladder (NZYDNA ladder V) (9).
